# Oxidative stress: an evolving definition

**DOI:** 10.12703/r/10-13

**Published:** 2021-02-09

**Authors:** Li Li Ji, Dongwook Yeo

**Affiliations:** 1 The Laboratory of Physiological Hygiene and Exercise Science, School of Kinesiology, University of Minnesota Twin Cities, Minneapolis, Minnesota, USA; 2 Department of Orthopedic Surgery, Mayo Clinic, Rochester, Minnesota, USA

**Keywords:** free radical, mitochondria, oxidative stress, redox signaling, skeletal muscle

## Abstract

Thirty-five years ago, Sies and colleagues insightfully described the universal phenomenon that the generation of reactive oxygen species could modify macromolecules in living organisms, resulting in a wide range of measurable damage. They used the term “oxidative stress” to define the loss of the balance between oxidants and antioxidants in favor of the former. After decades of research, it became increasingly clear that cells are not simply passive receivers of oxidative modification but can act dynamically to resist and adapt to oxidants. Furthermore, many redox-sensitive pathways have been identified wherein certain oxidants (mainly hydrogen peroxide and nitric oxide) are used as messenger molecules to transduce the signals required for these adaptations. Since the turn of the century, redox signaling has developed into a vibrant multidisciplinary field of biology. To reflect the evolution of the study in this field, the definition of oxidative stress is postulated to define a state in which the pro-oxidative processes overwhelm cellular antioxidant defense due to the disruption of redox signaling and adaptation.

## Introduction

Thirty-five years ago, when Sies and Cadenas^1^ in their landmark paper “Oxidative stress: damage to intact cells and organs” first introduced the terminology of oxidative stress, few people realized the impact of this contribution, while the definition was still a rather vague one. During the past several decades, free radical chemistry has developed far beyond a subfield of chemistry and becomes a widely known major interdisciplinary field among chemistry, biology, and medicine^2^. Increasing numbers of researchers have now applied the definition of oxidative stress to describe a state of disturbance, damage, and pathogenesis caused by reactive oxygen and nitrogen species (RONS) in their respective areas. Despite tremendous progress, the concept of oxidative stress is still evolving. Therefore, the purpose of this short communication is to review the historical aspects and the current status of the background of its path of evolution.

## Historical definition

In their 1985 paper, Sies and Cadenas^1^ described a wide range of evidence wherein free radicals could be detected by various biomarkers and instrumentation and were shown to lead to cellular damage. Importantly, they pointed out that oxidative damage could result not only from external free radical insults such as irradiation and toxic chemicals but also from internal cellular mechanisms such as hydroperoxide production via normal metabolism, mono-oxygenase activation in response to xenobiotics, aldehyde formation, singlet oxygen generation, and glutathione (GSH) redox exchange^3^. Six years later, Sies formally gave the definition of oxidative stress as “a disturbance in the pro-oxidant-antioxidant balance in favor of the former”^4^.

Traditionally, oxidative stress has been defined by four categories of biological changes^2^. The first category is the detection of RONS generation in the cell. Here, RONS can be in either their radical forms (i.e. with unpaired electrons), such as superoxide, hydroxyl radicals, peroxyl radicals, and nitric oxide, or their non-radical forms, such as hydroperoxide, singlet oxygen, ozone, and peroxynitrite^5^. The second category describes the changes (usually the decrease) in cellular antioxidant defense capacity. This may be caused by declined abundance of low-molecular-weight antioxidants such as α-tocopherol (vitamin E), ascorbic acid (vitamin C), GSH, and carotenoids owing to either dietary deficiency, depletion by RONS, or both. Decreased activity and/or protein content of antioxidant enzymes, such as superoxide dismutase (SOD), catalase, glutathione peroxidase (GPX), and their auxiliary enzymes such as GSH reductase (GR), glucose 6-phosphate dehydrogenase, and isocitrate dehydrogenase, can also result in oxidative stress. A number of reasons can contribute to the decline of antioxidant enzyme defense, such as gene mutation or knockout, prosthetic metal ion deficiency, drug inhibition, or simply organism aging^6^. Interestingly, some organs and tissues may increase antioxidant enzyme expression to protect against RONS and thus be indicative of oxidative stress. An example is increased antioxidant enzyme activities in skeletal muscle with aging^7^. The third category pertains to biomarkers of oxidative stress, often termed fingerprints or footprints, reflecting the oxidative modification of a range of macromolecules. The most widely used biomarkers include lipid peroxidation (usually measured by lipid peroxide, isoprostane, 4-hydroxynonenal, and malondialdehyde), protein oxidation (measured by carbonyl formation and individual amino acid oxidative modification, such as hydroxyl- and o-tyrosine, methionine sulfoxide, and 2-oxohistidine), and DNA oxidation due to the formation of 8-hydroxyl-2’-deoxyguanosine^5^. Finally, a disturbance of the cellular redox status is also regarded as evidence of oxidative stress^1^. Altered GSH to glutathione disulfide (GSSG) ratio and reduced-to-oxidized thioredoxin (TXN) ratio are reliable indications of redox changes^2^. It is noteworthy that redox status changes are closely related to other categories of oxidative stress. For example, a surge of RONS production can overwhelm the GPX-GR cycle’s reductive capacity, resulting in a rise of GSSG level with subsequent efflux from organs and tissues^8^. Moreover, decreased GSH/GSSG and TXN ratio can render some enzymes in the signal transduction pathways inactive, thus facilitating RONS formation and even causing redox signaling disruption (see below).

Inflammation, either acute or chronic, taking place either in the muscle cell or in the joints and soft tissues represents another form of disturbance of the balance between oxidants and antioxidants and may lead to oxidative stress^9,10^. Inflammation reflects the response of the organism to invading pathogens or internal damage as an adaptive protection. Initial infiltration of plasma polymorphoneutrophil (PMN) into the affected tissues can promote superoxide generation by NADPH oxidase located on PMN membrane, with subsequent production of superoxide, hydroperoxide, and hydrochloric acid to “disinfect” the damage site^2^. However, excessive inflammation may be inflicted by overproduction of pro-inflammatory cytokines such as tumor necrosis factor (TNF) α and interleukin (IL)-1 and -6 and activating nuclear factor (NF) κB pathway^11^. Escalation of local inflammation due to NFκB activation can lead to elevation of plasma C-reactive protein (CRP), a liver-derived protein widely regarded as a biomarker of systemic inflammation^10^. Chronic inflammation within muscles and ligaments after initial injury may attract PMN and macrophages to the injury sites and further enhance RONS production, forming a vicious cycle^12^.

Although the above categories of oxidative stress markers are widely used and effective, there are limitations in elucidating the details and nature of the cellular events. The most significant limitation of the early definition is that it somewhat implies that the biological system is a passive receiver of oxidative damage and the effects of RONS on the macromolecules are stoichiometrical^3^. Research advances, especially after 1990, have gradually revealed that this is not the case. The discovery that some antioxidant enzymes can adapt to elevated reactive oxygen species (ROS) generation under various pathological, physiological (such as exercise and aging), and nutritional (such as certain metal ion deficiency or overload) conditions suggests that organisms can alter their internal resistance to RONS and oxidative insult to reach a new balance, i.e. oxidative-antioxidant homeostasis^2^. Thus, oxidative stress may mean that the system has failed to adapt to or resist the oxidants or, in the case of inflammation, has over-reacted to the initial oxidative insult. Thus, in 2006, Jones^13^ proposed modifying the definition of oxidative stress to “a disruption of redox signaling and control”. While this proposed new concept recognizes the dynamic nature of the oxidative-antioxidant balance, it was not widely appreciated until the mechanisms by which certain ROS can serve as signaling agents to modulate the antioxidant system and even metabolic functions were fully uncovered.

## Role of redox signaling

Cell signaling (also termed signal transduction) is one of the three means (other than hormones and synapsis) by which cells respond to external stimulus, via transient allosteric or covalent protein modifications or change of gene expression. Redox signaling defines a process in which cells use certain RONS as the signaling molecules for transformation and differentiation^11^. The role of cysteine sulfhydryl, serving as a critical moiety on the active site of glutamine synthetase, was an early example of how redox balance of a single amino acid can affect a specific cell function^1^. The discovery that the gene *OxyR* could respond to the oxidative environment by expressing a particular gene product may be viewed as the simplest form of redox signaling^14^. Over several decades of research, it is now well established that a wide range of cellular functions including growth, adaptation, and senescence all involve redox signaling^11^. According to Lander^15^, at least five categories of cellular responses can be stimulated by ROS, including the modulation of cytokine, growth factor, or hormone action and secretion; ion transport; transcription; neural modulation; and apoptosis. Although early research paid much attention to the role of redox changes of protein sulfhydryl in the regulation of cell function^16^, more recent studies indicate that nearly half of the above-mentioned effects require members of the NFkB and mitogen-activated protein kinase (MAPK) pathways to participate^11^. Furthermore, the redox regulation of protein tyrosine phosphatase (PTP) by ROS has received great attention in redox signaling^17^. Permanent activation of PTP due to an overwhelming reductive environment may render the enzymes bearing PTP incapable of responding to redox changes and making functional adjustments.

But why does a disturbance or loss of control of redox signaling, as suggested by Jones^13^, imply the occurrence of oxidative stress? First, redox signaling depends, to a large extent, on the generation and a stable concentration of RONS. Allen and Trensini^11^ summarized hundreds of studies in which ROS were used as signaling messengers and found that over half of the cases were conducted by H_2_O_2_. If the source of ROS is dramatically disturbed, redox signaling will not function properly. For example, the steady state H_2_O_2_ concentration in the mitochondrion is dependent on the coordinated actions of MnSOD (SOD2) and GPX; genetic deficiency or gene knockout of MnSOD can lead to superoxide spill, which reacts with H_2_O_2_ to produce highly reactive hydroxyl radicals, resulting in cell necrosis or death^5^. Conversely, an overwhelmingly reductive environment, such as an excessively high NADH/NAD^+^ or GSH/GSSG ratio, can also destroy redox signaling and result in “reductive stress”^18^. Second, most antioxidant enzymes contain gene regulatory sequences in their promoter and intron regions that can interact with redox-sensitive transcription factors to trigger upregulation of gene expression^19^. Failure to increase antioxidant defense in the face of increased oxidant production can render the cells susceptible to oxidative damage. For example, the genes of SOD2, GPX1, inducible nitric oxide synthase (iNOS), and glutamylcysteine synthetase (GCS, the rate-limiting enzyme for GSH synthesis) contain conserved sequences for NFκB binding^11^. Rats treated with allopurinol to supress superoxide and H_2_O_2_ production via xanthine oxidase were shown to fail to upregulate SOD2, iNOS, and PGC-1α in response to endurance training^20,21^. Third, there is evidence that the decline of normal cell functions due to muscle immobilization^22^, diaphragm passive ventilation^23^, muscle ischemia^24^, and aging^25^ can also trigger oxidative stress, and a major cellular mechanism may involve the discord of redox signaling. Loss of mitochondrial homeostasis (controlled by mitochondrial biogenesis, morphological dynamics, and mitophagy) leading to increased ROS release, downregulation of antioxidant defense, and inflammation has been implicated as a potential mechanism for the disturbed redox signaling in skeletal muscle^26,27^. Although MAPK and NFκB are the most studied, other signaling pathways have been increasingly identified to be sensitive to redox disturbances, such as PGC-1α^28^, AMPK^29^, FoxO family transcription factors^30,31^, and sirtuins^32,33^. In this regard, failure of proper redox signaling as a potential mechanism for aging is of particular interest to the scientific community and deserves a more extensive review^34^.

## Oxidative stress and hormesis

Another way to define the delicate relationship between oxidative stress and redox signaling is hormesis. Hormesis is now widely recognized to be a universal phenomenon to describe an organism’s response to stress, displaying a dose-response curve^35^ that resembles an inverted U^36^. The dynamic of hormesis implies that a stress below the “breaking point” (i.e. the peak of the curve) may elicit a positive response that would make the organism more resistant to higher levels of stress. Hormetic concept has been applied to several subdisciplinary studies such as hypothermia, heat, ischemia, starvation, pro-oxidants, and other types of stress such as pain, sleeplessness, noise, cold^37,38^, and exercise^39^.

Examples that illustrate cellular adaptive response to oxidative stress are numerous. Physical exercise represents a unique stress that animals encounter in order to maintain mobility, seek food, and ensure reproduction and survival through rigorous muscle contraction, leading to increased ROS production^6,40^. Several exercise physiologists pioneered the idea that ROS might stimulate the body’s antioxidant defense and either reduce ROS production or increase their removal, or both^41^. The discovery that H_2_O_2_ could induce gene expression of antioxidant enzymes via NFκB in muscle cells provided unequivocal biochemical and molecular biological mechanisms of how such adaptations might occur^42^. Radak *et al*.^43^ and Ji *et al*.^44^ postulated that hormesis might underlie the cellular mechanism of exercise adaptation of antioxidant defense that is stimulated by ROS generation. It is now clear that while the cellular antioxidant system maintains a basal “reserve” to handle low-level oxidative challenge, most antioxidant adaptation requires *de novo* protein synthesis through transcription, translation, post-translational modification and protein transport^40,45,46^. Over the past few decades, new research has revealed that hormesis takes place in a wide range of biological activities, including but not limited to increased mitochondrial biogenesis^26,47,48^, change of mitochondrial morphology through fusion and fission dynamics^49^, and enhanced mitophagy to degrade damaged organelle^50,51^. During these hormetic responses to oxidative stress, redox signaling plays a vital role.

## Conclusion

**Figure 1.**
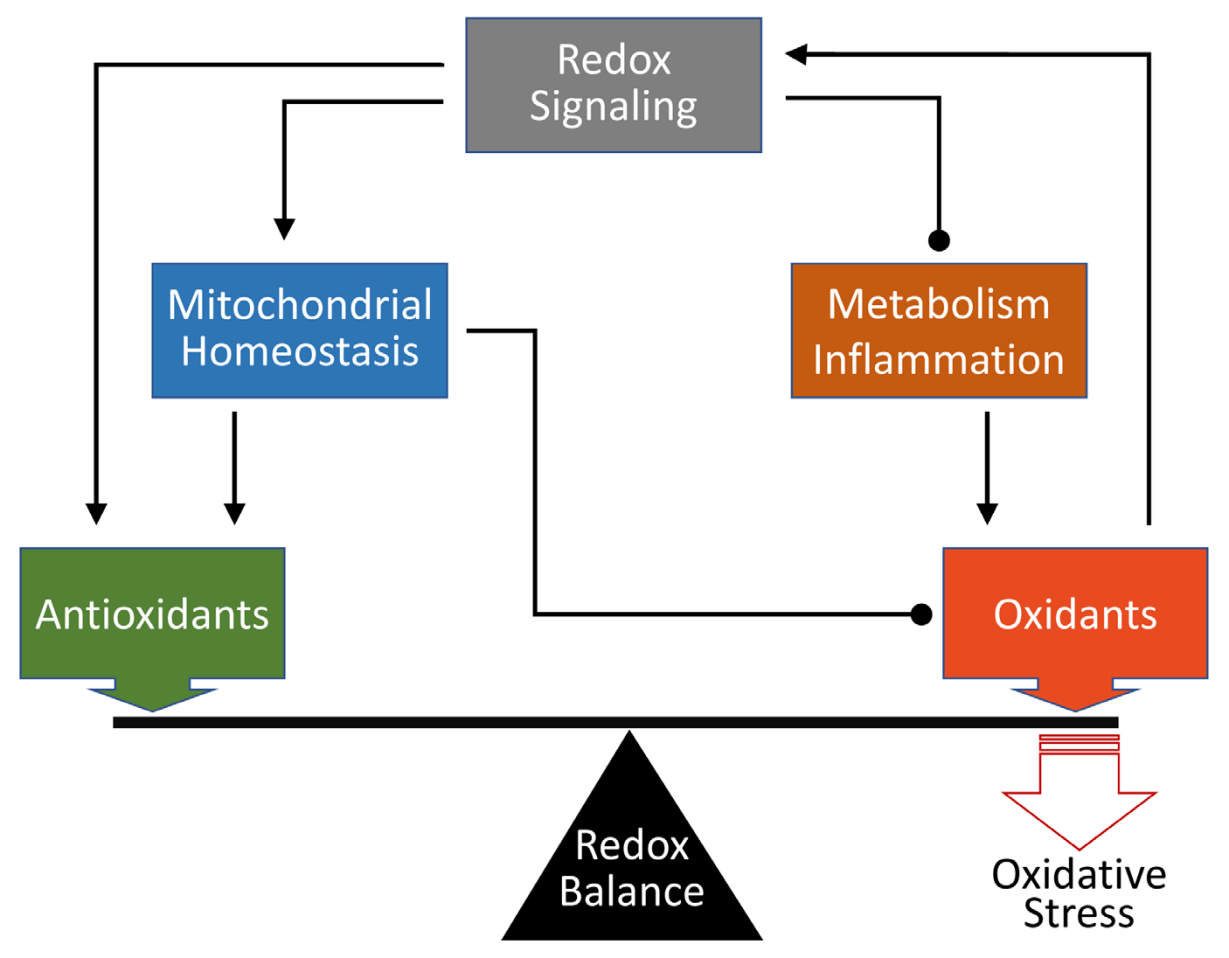
Schematic representation of the roles of oxidants and antioxidants in determining oxidative stress. Through redox signaling, oxidants can directly upregulate antioxidant defense or indirectly ameliorate mitochondrial homeostasis, thus reducing oxidant production. Redox signaling also modulates cellular metabolic process and inflammation to minimize oxidants. Failure or discord of redox signaling leads to a tip of oxidant-antioxidant balance in favor of the former and thus oxidative stress. Arrow-headed lines represent activation; dot-ended lines represent inhibition.

The generation of RONS is an inevitable process in aerobic life^5^. According to the Second Law of Thermodynamics, organisms need to obtain energy substrates from the environment to generate ATP for repair, growth, and reproduction, whereas the costs of converting nutrients to low-entropy forms for self-preservation are the production of high-entropy wastes (water, CO_2_, urea) and heat^52^. Throughout life, the balance between the oxidative process to release energy from substrates and the antioxidant defense to minimize self-destruction continues. Thus, oxidative stress is inevitable and has been postulated as the most important biological mechanism for aging^53–55^. Meanwhile, the definition of oxidative stress has evolved from a more descriptive term emphasizing end results to a more dynamic alternative reflecting the role of adaptive processes involving redox signaling and hormesis^45^. At the end of the day, the authors favor a modified definition to summarize the points of view herein: oxidative stress defines a state in which the pro-oxidative process overrides cellular antioxidant defense due to the disruption of redox signaling (Figure 1).
